# Dispersal and Migration Patterns of Freshwater Semiaquatic Bugs

**DOI:** 10.3390/insects12110976

**Published:** 2021-10-28

**Authors:** Tomáš Ditrich

**Affiliations:** Faculty of Education, University of South Bohemia, 37005 Ceske Budejovice, Czech Republic; ditom@pf.jcu.cz; Tel.: +420-387-773-014

**Keywords:** gerromorpha, water striders, water cricket, gerridae, veliidae, flight dispersal, trade-off, wing polymorphism

## Abstract

**Simple Summary:**

Semiaquatic bugs have colonized the water surface of most aquatic habitats, from small and temporary puddles and streams to the open ocean. Most semiaquatic bugs are wing-polymorphic, as some individuals have fully developed wings (macropterous) and others have shortened wings (brachypterous) or are wingless (apterous). This is characteristic for most temperate water striders, common on lakes, fishponds, and pools around the world. The report presented here is based on the collection of more than 23,000 individuals of nine species of semiaquatic bugs that were individually marked by a unique code and released. The recaptures revealed several distinct dispersal strategies, with differences among individual species. Collection of the marked bugs also helped to describe dispersal via the water surface by flightless individuals. Using the results of the presented survey, our knowledge of these interesting insects is considerably extended, and it can help us to understand the general dispersal patterns of aquatic insects.

**Abstract:**

Semiaquatic bugs (Hemiptera: Heteroptera: Gerromorpha) are mostly wing-polymorphic species with flight dispersal as an important life history trait, but the specific flight ability and dispersal pattern remain unexplored in most species. This report presents the results of a long-term survey based on the individual marking of more than 23,000 specimens of eight water striders (Gerridae) and a water cricket *Velia caprai* (Veliidae). Three distinct lentic habitats were sampled (solitary fishponds, systems of nearby fishponds and systems of small, often temporary pools) and one lotic habitat—a small forest stream. Recaptures revealed that three gerrid species tend to stay at the breeding site, but can differ in dispersal via the water surface. Reproductive flightless females disperse most actively via the water surface, possibly bypassing the trade-off between dispersal and reproduction. One species has a sex-dependent dispersal pattern, with females being rather philopatric, whereas males often disperse. Three other gerrid species are highly dispersive and tend to change breeding site. *V. caprai*, the only lotic species included in this survey, tend to move upstream and possibly compensate for the downstream drift.

## 1. Introduction

Aquatic insects have surprisingly good dispersal abilities, as many natural freshwater habitats are more or less temporary. The dispersal and migration capabilities of aquatic insects have thus been reviewed several times [1,2]. Generally, aquatic insects often migrate to and from an overwintering habitat (autumn and spring in the temperate zone) and disperse during the breeding season. The dispersal of aquatic insects can result in the colonization of new habitats, but often occurs in response to harsh conditions in the habitat. These harsh conditions, acting as cues for dispersal, can include the drying out of water bodies, high population density, or the presence of a predator (reviewed by [1]).

Among the aquatic insects, semiaquatic bugs (Hemiptera: Heteroptera: Gerromorpha) deserve special attention. They occupy the water surface and littoral zone [3], and can be considered as a transitional between truly aquatic and terrestrial insects, as they live in close proximity to water (usually on the water surface) but never or rarely submerge [4,5]. Many gerromorphan species are wing-polymorphic—they can be macropterous (long winged, LW), brachypterous (short winged with varying wing length, SW), or apterous (wingless) (Figures S1–S4). Generally, macropterous individuals are considered as being adapted for dispersal, whereas brachypterous and apterous individuals do not disperse—such alary polymorphism can be regarded as an example of adaptive morphology for dispersal [1]. The proportion of wing morphs can be even used as an ecological indicator [6]. The dispersal flight by semiaquatic bugs is thus an important life-history trait [3], but the exact extent of migration and dispersal has rarely been studied, with the exception of a long-term survey of the seasonal aspects of flight in five Canadian water striders by Spence [7]. He identified four distinguished categories of seasonal flight activity, connected with overwintering sites and intersite movement. Two *Gerris* spp. were also caught during spring dispersal flight in Sweden [8]. Here, I present the results of a long-term study of nine gerromorphan species focused on individual migration and dispersal patterns of both inter- and intrasite movement.

## 2. Materials and Methods

Mark-recapture of individual specimens was used in this research. The pronotum of each specimen was first painted with transparent nail varnish. A tag with a code (photographic paper with a printed combination of a number and a letter (printer Canon IR2018, font size no. 2 and 3) was placed on the pronotum and repainted using varnish for the second time (Figures S1 and S4). This method, together with results of survival and effect on flight capability, was described in [9] in detail. The adult insects were caught in the spring (years differed in particular sites, see below for details) soon after first appearance (usually during April), individually marked and released. This was repeated three times, usually with a one-week interval. The sites were then regularly sampled every three weeks without marking new individuals (just recording the marked ones). During August, when the overwintered generation had vanished, the new generation adults were marked again during three subsequent samplings in the one-week interval. After that, the sampling without marking of the new individuals continued at three-week intervals until November, when the insects disappeared. Every site was sampled for at least two full subsequent seasons. Marking of the adults was finished after the April of the last sampling season, since only already marked individuals were recorded.

Three types of lentic habitats were chosen for the survey of dispersal and migration patterns: (a) two solitary fishponds for the description of the within site dispersal (all adult gerrids were sampled); (b) two fishpond systems for the description of intersite dispersal (only macropterous adult gerrids were sampled); and c) two small pool systems for description of dispersal in harsh conditions (all adult gerrids were sampled).

1. Solitary fishponds, sampled from April 2009 to November 2011, included:
  - Zavratsky fishpond (48°56′31.547′′ N, 14°22′59.948′′ E), about 8 km southwest of Ceske Budejovice (South Bohemia, Czech Republic) and ca. 2.2 km away from the next closest fishpond. It is a roughly round fishpond ca. 300–390 m wide, surrounded by a row of trees and meadows. The littoral zone is relatively well-developed, i.e., aquatic vegetation (including floating leaves) is dense, present along 60–70% of the shore. The shore was divided into seven sampling sites (sectors) (usually 40–60 m long) concerning natural borders (fallen logs, shore topography, etc.). Every sampling site was sampled for 10 minutes using a hand net (40 cm diameter; ca. 2 m handle), and determining, marking and counting the bugs took place after sampling. Sampling was performed either from the shore or from an inflatable boat (some parts of the shoreline are not accessible from land). The movements of the recaptured bugs were scored as 0 (recaptured at the same site), 1 (recaptured at an adjacent site), 2 (recaptured at a site over one site away), or 3 (recaptured at a more distant site over two sites away). See Figure S5 for the landscape position. An aerial view and position of the seven sampling sites of the fishpond with an example of the movement scoring can be found in Figure S6.
  - Kamenny fishpond (49°9′24.077′′ N, 14°31′43.604′′ E), about 20 km north of Ceske Budejovice and ca. 1.6 km away from the next closest fishpond. It is a roughly round fishpond ca. 280–330 m wide, surrounded by a row of trees/bushes and meadows. The littoral zone is well-developed. Sampling and scoring were set equally, as in the previous fishpond. See Figure S7 for the landscape position. An aerial view and position of the seven sampling sites of the fishpond can be found in Figure S8.

Flight ability (capable of flight—macropterous; or flightless—brachypterous and apterous) was also recorded, and the proportion of all marked macropterous adults was compared with the proportion of macropterous recaptured adults. This allowed for monitoring eventual changes in this parameter. The proportion of macropterous individuals was also recorded in November 2010 (last sampling before the winter) and April 2011 (first sampling after the winter). A comparison of these two proportions could show if both flightless and macropterous individuals return to the site after overwintering.

During 2017, additional sampling at these sites was performed. Females of *Aquarius paludum* and *Gerris lacustris*, marked from May to September, were dissected upon recapture and the state of their ovaries was checked. The reproductive state of females was evaluated as a) reproductive (ovaries with chorionated eggs or developing eggs, or oviposition indicated by rest of follicle epithelium) or b) non-reproductive (undeveloped ovaries, without mature oocytes). Wing morphs were also recorded.

1. Fishpond systems, sampled from April 2007 to November 2008 (system a) and from April 2011 to November 2012 (system b), included:
  - Bily Kamen fishpond system—This is a system of eight fishponds (Bejchlin, Bejchlinec, Bily Kamen, Hluboky, Slatinka, Vezisko, Veziste, and Noname fishpond), creating a rough line, where every fishpond is a maximum of 150 m away from two other fishponds. The distance between the two most distant fishponds (edge to edge) is ca. 930 m. The whole fishpond system is mainly surrounded by fields and marshes. The central fishpond (Bily Kamen, 49°20′33.560′′ N, 13°46′50.431′′ E) is located about 65 km northwest of Ceske Budejovice. The littoral zone is highly variable, being well-developed in five fishponds and poor in three others. For sampling, every fishpond was divided into 3–9 sampling sites (according to the size of the fishpond; only places with great natural gerrid aggregations were sampled). The number of fishpond changes was recorded for every recaptured bug. The landscape position of the fishponds can be found in Figure S9.
  - Motovidlo fishpond system—This is a system of seven fishponds (Motovidlo, Vysatov, Maly Machovec, Horni Machovec, Dolni Machovec, Mlynsky, Blatec), creating a rough circle, where every fishpond is a maximum of 200 m away from two other fishponds. The distance between the two most distant fishponds (edge to edge) is ca. 900 m. The whole fishpond system is mainly surrounded by fields, and there is a forest in the middle of the fishpond system (49°0′6.971′′ N, 14°22′39.981′′ E) about 7.5 km northwest of Ceske Budejovice. The littoral zone is highly variable, being well developed in three fishponds and poor in four others. Sampling and scoring were set equally, as in the previous fishpond system. The landscape position of the fishponds can be found in Figure S10.
2. Small pool systems, sampled from April 2013 to November 2014, included two systems of artificial pools in a sandpit:
  - Cep I—This is a system of 12 small pools in the Cep I sandpit (48°55′4.473′′ N, 14°52′55.670′′ E) about 30 km east of Ceske Budejovice. Every pool is roughly round and ca. 3–4 m in diameter (only one is larger, roughly an ellipse 20 m long and 6 m wide), and the pools are regularly placed ca. 5–7 m from each other. The pools are less than 1 m deep, mostly with prevalent aquatic vegetation (both emergent and floating). Surrounding the pools is mainly bare sand. During sampling, virtually all adult gerrids were sampled. Because the recapture rate was relatively low in these systems (see below), for every recaptured insect, it was recorded whether it had stayed or moved from the last capture and whether this last capture was at a pool with or without vegetation. All pools were fed with water throughout the sampling seasons, but the water level was sometimes low. An aerial view of the pools can be found in Figure S11.
  - Cep II—This is a system of more than 35 small pools in Cep II sandpit (48°54′52.988′′ N, 14°52′29.647′′ E) about 30 km east from Ceske Budejovice (located 650 m southwest from the previous pools). Every pool is roughly round and ca. 3–4 m in diameter, and the pools are regularly placed ca. 3–5 m from each other. Pools are less than 1 m deep, mostly without aquatic vegetation. Surrounding the pools is bare sand. Sampling and scoring were set equally as in the previous pools system. About 2/3 of the pools were fed with water throughout the sampling seasons, whereas some of the pools were temporary. An aerial view of the pools can be found in Figure S12.

As a lotic habitat, two forest streams in Novohradske Mountains. with abundant water cricket *Velia caprai* (Heteroptera: Gerromorpha: Veliidae) populations were chosen. The first stream is near Pohori na Sumave village (48°36′59.763′′ N, 14°39′31.126′′ E), a small stream (usually 30–130 cm wide, maximal depth 30 cm) in a spruce forest (described in detail in [10]). In the sampling area, there are two smaller streams with a confluence into one stream. About 130 m of the first and 90 m of the second branch were divided into 29 and 17 sampling sites, respectively. The sites were distinguished by the natural borders (small rapids, weirs or places with great stream velocity, Figure S13). Either the upstream or downstream distance (the distance passed between first capture and last recapture) was scored for recaptured individuals. The streams were sampled from October 2008 to November 2010, and the streams were also searched for marked individuals up to ~200 m upstream and downstream from the sampling area. The marking scheme, distinguishing overwintered and a new generation of the gerrids, could not be used for *V. caprai*, because it is a species which is able to survive two subsequent winters as an adult [11,12]. The overwintered adults were marked from April to June (marking was ceased when 4th or 5th instar larvae appeared, usually in June), the site was sampled with two week intervals, and all adults caught were marked. For marking the new (summer) generation adults, the 3rd–5th instar larvae of *V. caprai* were captured and allowed to develop in plastic boxes close to the original sampling site. The newly eclosed adults were marked and released at the original larval site. This was repeated during the period of June–September 2009–2010.

The second sampling stream is about 2 km north (48°38′13.299′′ N, 14°39′46.132′′ E) and is also a small stream in a spruce forest. The sampling and scoring of the recaptures were the same as in the previous stream.

### Data Analysis

Movement in solitary fishponds (within-site dispersal) was first analyzed using the general regression model (GRM), evaluating the number of sectors moved from last capture (0–3; dependent variable), with species, sex, wing morph, and all their combinations as factors. Forward stepwise regression was used for finding significant factors. In the case of a significant effect of species, the data were analyzed separately in particular species by factorial ANOVA (sex and wing morph as factors). The same procedure was applied for movement analysis of reproductive vs. non-reproductive females (reproductive state replaced sex as a factor). Comparison of a proportion of macropterous individuals between comparable subpopulations (marked vs. recaptured individuals or populations before and after winter) was performed using the paired *t*-test. The intersite dispersal was analyzed similarly with species and sex as factors (only macropterous individuals were marked), and for detailed analysis within species, a non-parametric test (Kolmogorov–Smirnov two-sample test) was used because of the relatively low number of moved recaptured individuals. The difference between the number of individuals that moved or stayed in pools with and without vegetation (small pools systems) was tested using the χ^2^ test in contingency tables, and both sexes were pooled due to the low number of recaptured individuals. The dispersal of *V. caprai* in forest streams was analyzed by factorial ANOVA (sex and generation as factors) based on distance moved (upstream dispersal as positive, downstream ass negative distance). The tendency of *V. caprai* to move upstream during winter was analyzed by χ^2^ test (upstream vs. downstream/none movement). All analyses were run in Statistica 13.5 (Tibco Software Inc., Palo Alto, CA, USA).

## 3. Results

More than 23,000 specimens of *Aquarius paludum*, *Limnoporus rufoscutellatus*, *Gerris argentatus*, *G. gibbifer*, *G. lacustris*, *G. lateralis*, *G. odontogaster*, *G. thoracicus* (Gerridae) and *Velia caprai* (Veliidae) were individually marked during the survey.

### 3.1. Within-Site Dispersal at Solitary Fishponds

During the three seasons, 986, 1079, and 1120 individuals were marked at Zavratsky fishpond. The species composition was (all seasons pooled, i.e., 3185 individuals): *G. lacustris* (38%), *A. paludum* (32%), *G. argentatus* (21%), *G. odontogaster* (8%) and *L. rufoscutellatus* (<1%). A similar situation was found at Kamenny fishpond—we marked 1124, 1052, and 1245 individuals, and species composition (all seasons pooled, i.e., 3421 individuals) was: *G. lacustris* (37%), *A. paludum* (23%), *G. argentatus* (18%), *G, odontogaster* (12%), *L. rufoscutellatus* (7%), and *G. thoracicus* (3%). The recapture rate (a proportion of recaptured marked individuals) was highest in *G. lacustris* (0.31, resp. 0.3) (Table 1). For all analyses, the data from both fishponds were pooled.

GRM revealed that the number of sectors moved from the last capture was significantly affected by species; sex; wing morph; sex * wing morph and species * sex * wing morph (all *p* < 10^−9^). The whole model was highly significant (*p* < 10^−17^; R^2^ = 0.27). The within-site dispersal was thus analyzed by species.

**Figure 1:**
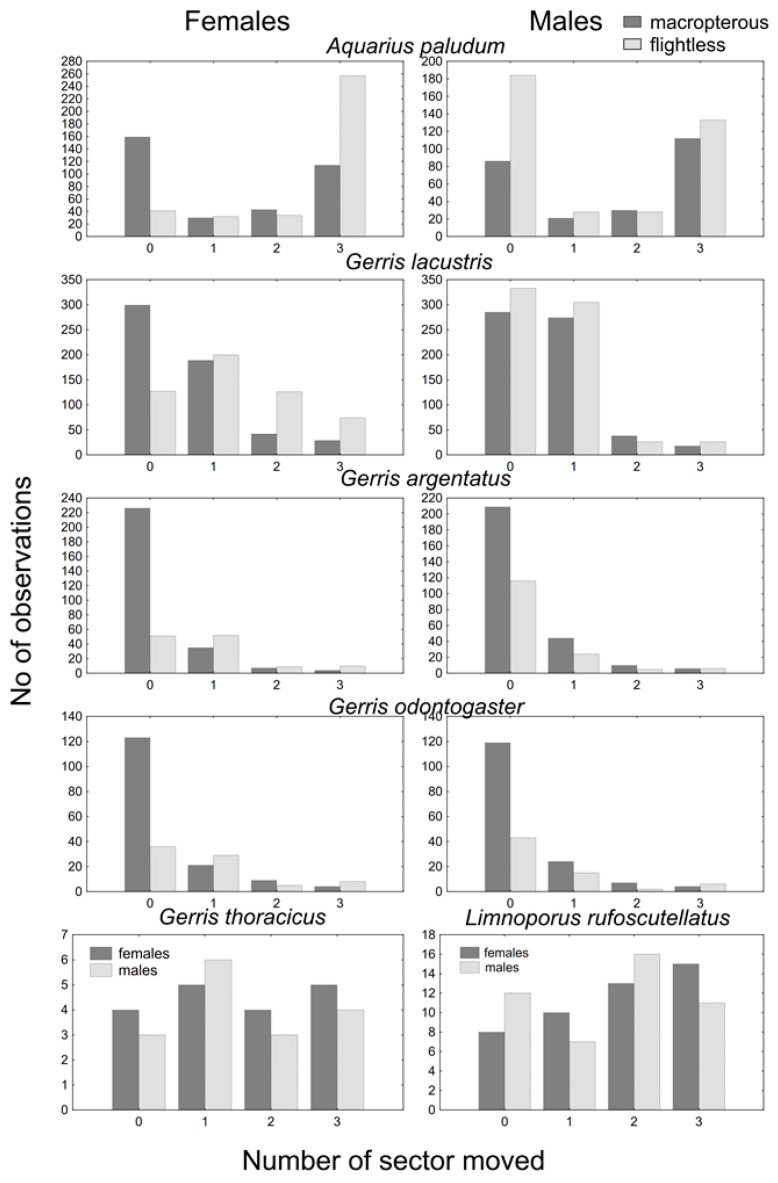
The score of within site movement. Both sexes and wing morphs are displayed for *A. paludum*, *G. lacustris*, *G. argentatus* and *G. odontogaster*; only macropterous bugs are shown for *G. thoracicus* and *G. gibbifer*.

**Figure 2:**
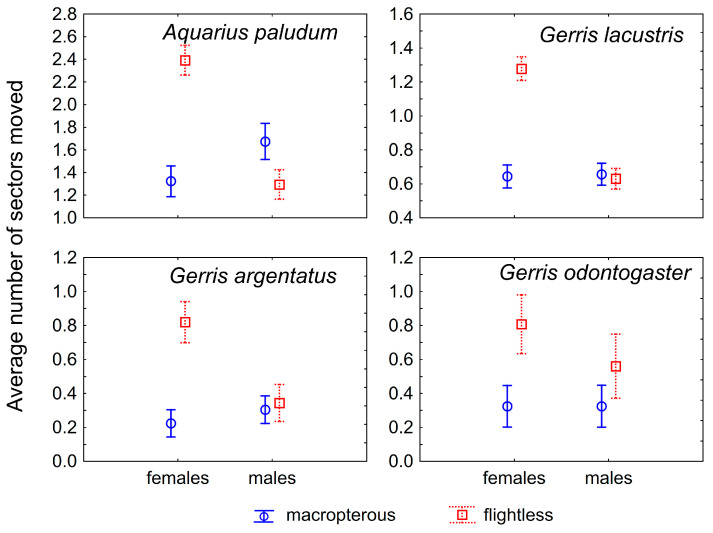
The mean of within-site movement in four wing dimorphic species; both sexes are displayed. Flightless females move significantly more than males and macropterous females. Vertical bars denote 0.95 confidence intervals.

The most common number of passed sectors was three in *A. paludum;* this pattern was evident in females, whereas males showed comparable cases of three and zero passed sectors (Figure 1). This holds especially for flightless individuals—the highest number of flightless females scored three (recaptured at the most distant sector), whereas flightless males usually scored zero (however, a score of three was the second most common; Figure 2). The effect of sex, wing morph, and their interaction was highly significant in *A. paludum* (Figure 2, Table 2).

*G. lacustris*, the most abundant species in both fishponds, also moved often, but the moved individuals usually scored one (moved to an adjacent sector; Figure 1). The tendency to move was similar to that observed in *A. paludum*—females moved more often than males, and the highest number of sector changes was recorded in flightless females (Figure 1 and Figure 2). All factors (sex, wing morph, and their interaction) affected the movement score significantly in *G. lacustris* (Table 2).

A slightly different pattern was found in *G. argentatus*. Most recaptured individuals stayed in the sector of the previous capture (Figure 1); however, again, flightless females moved more often than macropterous individuals (Figure 1 and Figure 2). All factors (sex, wing morph and their interaction) affected the movement score significantly in *G. argentatus* (Table 2).

Similarly, *G. odontogaster* tended to stay at the site of the previous capture (Figure 1). Although the tendency to move was highest in flightless females again (Figure 2), the movement of this species was significantly affected only by wing morph, as the flightless individuals moved more often than macropterous individuals.

Only macropterous individuals of *G. thoracicus* and *L. rufoscutellatus* were captured. These species shared a similar dispersal pattern—a similar number of all movement scores, without a significant sex-specific difference (Figure 1 and Figure 2, Table 2).

**Figure 3:**
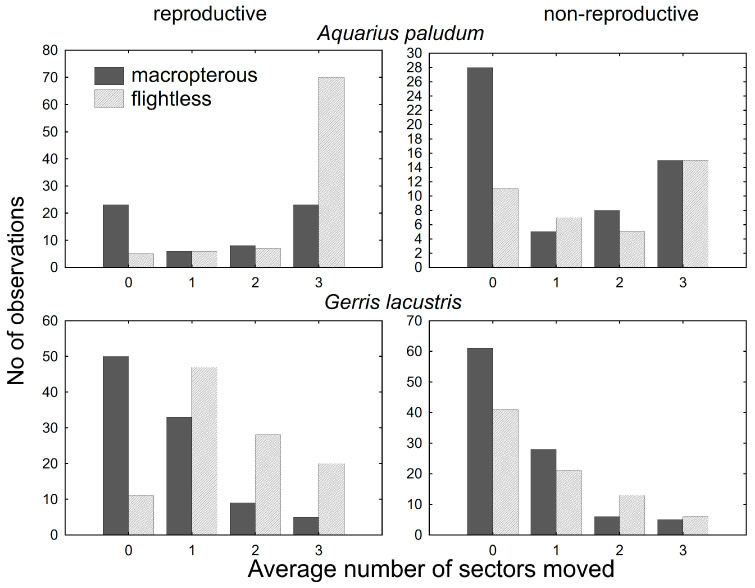
The score of within-site movement in wing-dimorphic reproductive and non-reproductive females of *A. paludum* and *G. lacustris*. In both species, the most dispersed are flightless reproductive females, although the number of passed sectors differs.

**Figure 4:**
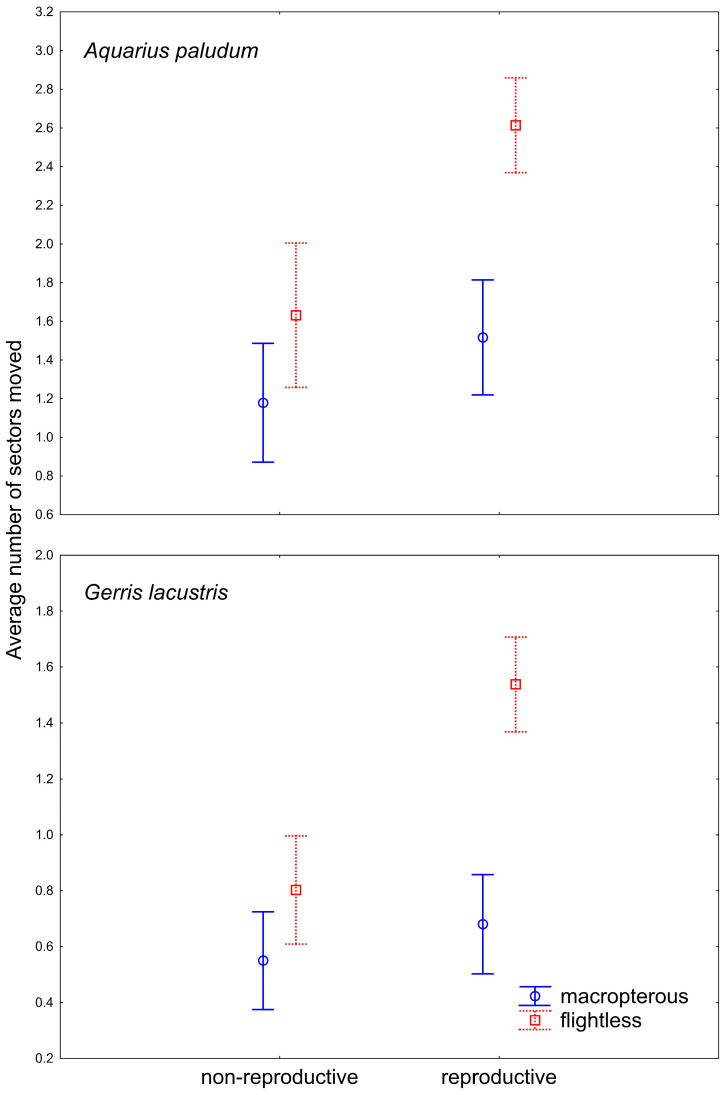
The difference in within-site movement in wing-dimorphic reproductive and non-reproductive females of *A. paludum* and *G. lacustris.* Vertical bars denote 0.95 confidence intervals.

For the movement analysis of females with emphasis on their reproductive state, 242 females of *A. paludum* and 384 females of *G. lacustris* were recaptured and dissected (Figure 3). GRM for the dispersal of females with different reproductive states revealed that the number of sectors passed through since the last capture was significantly affected by species, wing morph, reproductive state (all *p* < 10^−8^) and wing morph * reproductive state (*p* = 10^−4^). The whole model was highly significant (*p* < 10-^17^; R^2^ = 0.31). In both species, flightless reproductive females moved most often (Figure 4), despite the dispersal pattern differing between species. Flightless reproductive *A. paludum* females usually moved to the most distant sector of the fishpond, whereas *G. lacustris* females usually moved to an adjacent sector (Figure 3). According to factorial ANOVA, the effect of all factors and interactions was highly significant in both species (all *p* < 10^−3^), except for the reproductive state * wing morph interaction in *A. paludum*, with *p* = 0.04 (Table 3).

#### Proportion of Macropterous Individuals

**Figure 5:**
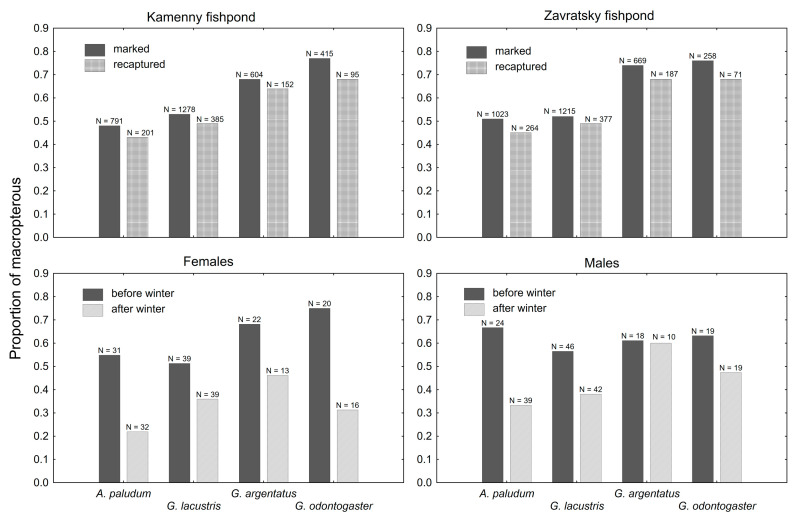
A comparison of the proportion of macropterous adults in marked and recaptured individuals at both sites (upper panels) and in both sexes before and after winter (bottom panels). The proportion of macropterous was lower in recaptured bugs (upper panels), and after the winter (bottom panels).

The proportion of recaptured macropterous individuals was lower in all wing-polymorphic species at both fishponds, when compared with the proportion of marked macropterous individuals (Figure 5). The difference was significant for both fishponds (t_3_ = 5.58; *p* = 0.01 at Zavratsky; t_3_ = 4.62; *p* = 0.02 at Kamenny fishpond). The proportion of macropterous individuals also decreased during winter (Figure 5) in both sexes of all wing-polymorphic species. The decrease was statistically significant in females (t_3_ = 4.58; *p* = 0.02) but not significant in males (t_3_ = 2.60; *p* = 0.08).

### 3.2. Intersite Dispersal at Fishpond Systems

The Gerromorpha species composition of both fishpond systems was similar, with seven and eight species and 6007 and 6075 marked macropterous individuals in combined seasons. The dominant species were *G. lacustris* and *A. paludum* in both fishpond systems (32–33%), followed by *G. argentatus* and *G. odontogaster* (11–15%). Other species—*G. thoracicus*, *G. gibbifer*, *G. lateralis*, and *L. rufoscutellatus*—were less abundant, with 0–6% of semiaquatic bug assemblages (Table 4). The recapture rate was between 0.11 and 0.35 (Table 4).

**Figure 6:**
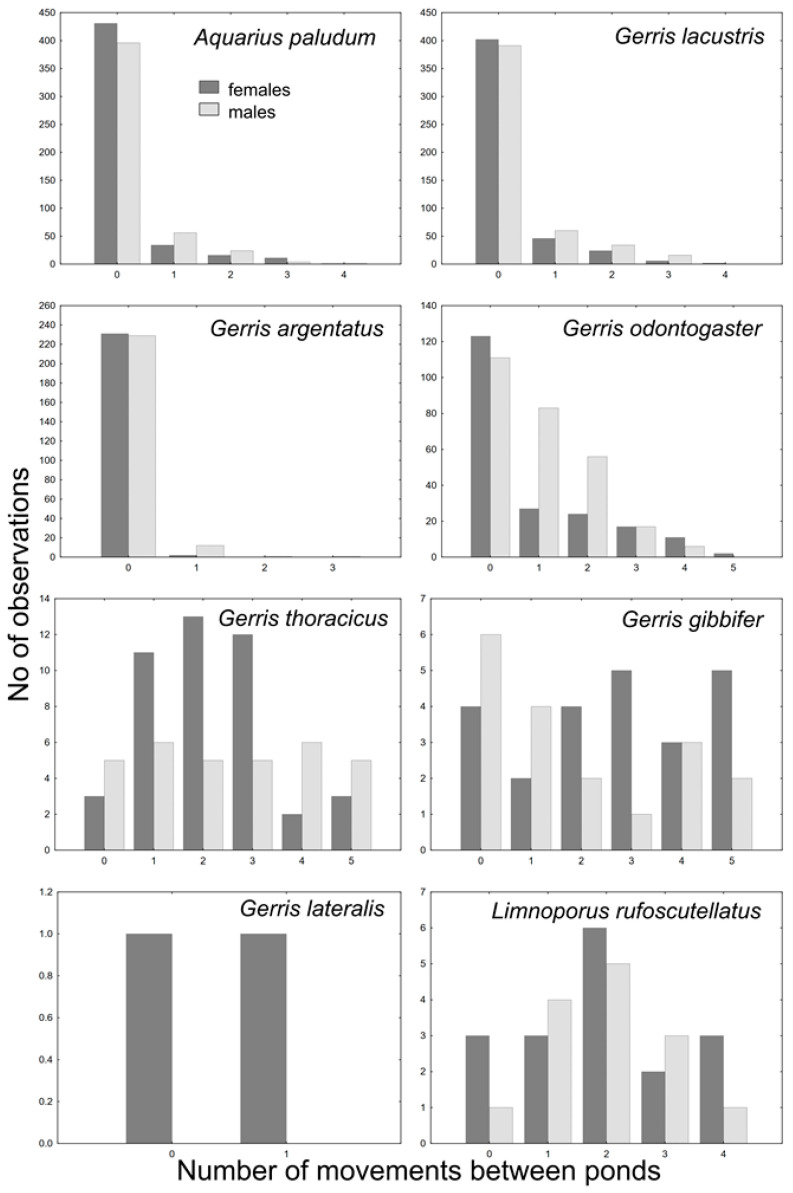
Movements between ponds in all recaptured species (intersite movements); only macropterous bugs were marked and recaptured.

**Figure 7:**
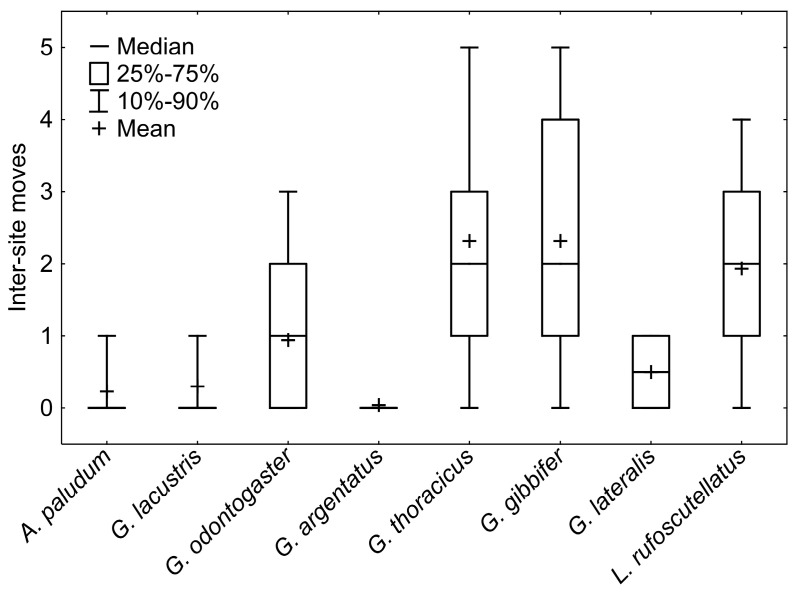
A comparison of intersite movements among particular species. *A. paludum*, *G. lacustris,* and *G. argentatus* are rather philopatric, *G. odontogaster* is intermediate, and *G. thoracicus*, *G. gibbifer,* and *L. rufoscutellatus* are frequent flyers. Only two individuals of *G. lateralis* were recaptured, and thus this species cannot be sorted into any of the above groups.

The GRM model of movement between ponds showed a significant effect of species (*p* < 10^−12^) and the interaction between species and sex (*p* < 10^−3^), and the effect of sex was marginally non-significant (*p* = 0.06). The whole model was highly significant (*p* < 10^−17^; R^2^ = 0.29). Dispersal pattern, differing among species, separates *A. paludum*, *G. lacustris* and *G. argentatus*, with a relatively low number of interpond movements, *G. odontogaster*, with males being more dispersed than females, and *G. thoracicus*, *G. gibbifer*, and *L. rufoscutellatus* dispersing among ponds irrespectively to sex (Figure 6 and Figure 7, *G. lateralis* was recaptured only twice with one movement). A significant effect of sex was found only in *G. odontogaster* (*p* < 10^−3^), whereas the effect of sex was not significant in all other species (all *p* > 0.1).

#### Remarkable Individual Records at Fishpond Systems

Two individuals of *G. thoracicus* (males, Bily Kamen fishpond system) were recaptured three times during summer 2007, with each recapture on a different fishpond. Together with the fishpond of original capture, they moved between at least four sites during the season. Similarly, one female of *L. rufoscutellatus* was present on at least three fishponds during 2008. Additionally, *G. gibbifer* (two males) moved between at least three fishponds (Motovidlo fishpond system) during summer 2012. During 2011, one male of *G. lacustris* and one male of *L. rufoscutellatus*, originally marked at the Zavratsky solitary fishpond, were recaptured at the Motovidlo fishpond system. The air distance between these two sites is ~6 km.

### 3.3. Dispersal at Small-Pools Systems

**Figure 8:**
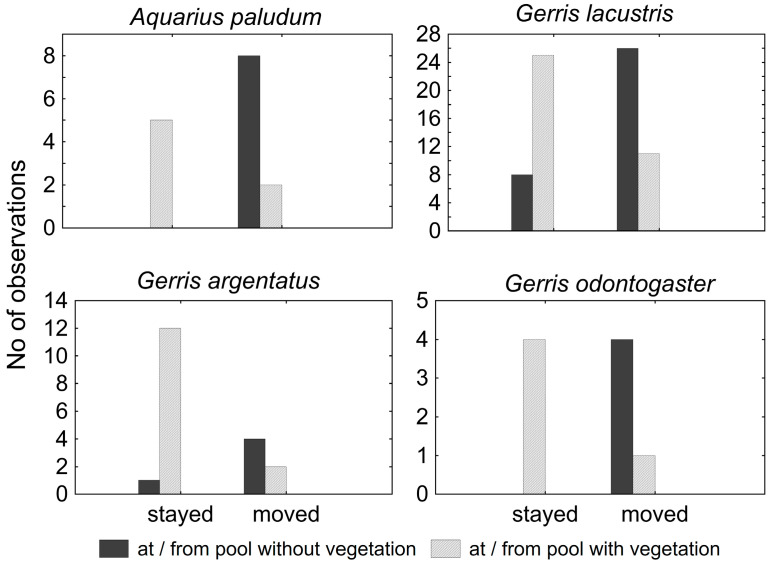
Water striders’ movements in small pools systems, concerning the vegetation (emergent and floating) of the pool. All species stayed in the vegetated pools but moved from the pools without vegetation.

The catches (all individuals were macropterous) at the small pool systems were relatively low, and all were during the period of April–June. During June in both sampling seasons, virtually all water striders disappeared. Nevertheless, the species composition was relatively rich in both sites, with five and seven species, respectively, but the recapture rate was relatively low (0–0.27) (Table 5). Because of the low number of recaptures in *G. thoracicus*, *G. gibbifer*, and *L. rufoscutellatus*, these species were omitted from analyses. Individuals showed a similar dispersal pattern in all remaining species—specimens from pools with vegetation rather tended to stay, whereas specimens caught at pools without vegetation usually moved to another pool (Figure 8). The difference between moving and staying in pools with or without vegetation was significant in all species (χ^2^ = 8.57, *p* < 10^−2^; χ^2^ = 14.79, *p* < 10^−3^; χ^2^ = 5.76, *p* = 0.02 and χ^2^ = 7.36, *p* < 10^−2^ for *A. paludum*; *G. lacustris*, *G. odontogaster*, and *G. argentatus*, respectively).

### 3.4. Dispersal by V. caprai at Lotic Habitat

**Figure 9:**
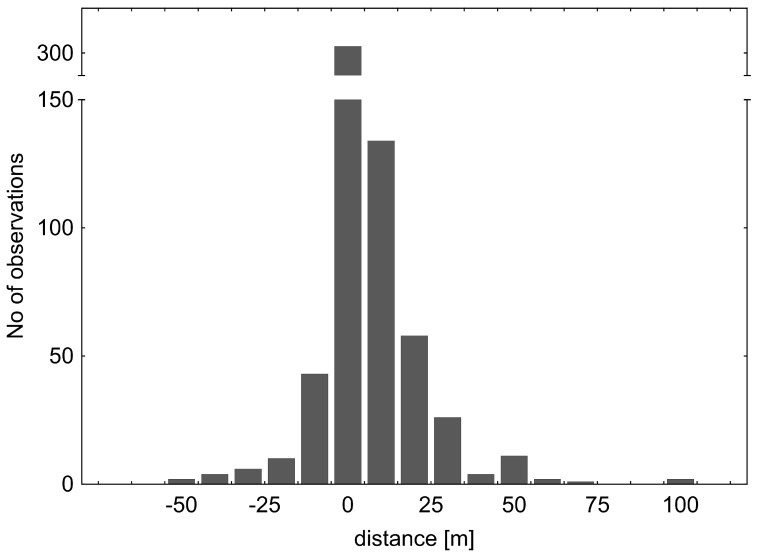
Total distance recorded for the water cricket *V. caprai*. Although most of the bugs stayed at the original site, more individuals moved upstream (positive numbers) than downstream (negative numbers). The upstream movement exceeded even 100 m.

**Figure 10:**
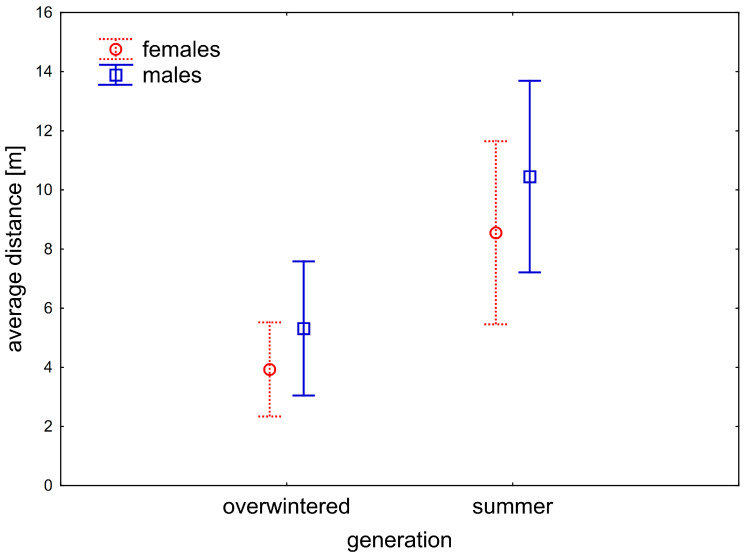
A relation of the movement and generation of *V. caprai*. The summer generation moves significantly further than an overwintered generation; this pattern is similar for both sexes. Vertical bars denote 0.95 confidence intervals.

Altogether, in all seasons and both streams, 1467 individuals of *V. caprai* were marked, and 606 individuals were recaptured (recapture rate 0.41; average recaptures = 1.55, most recaptures by an individual = 7). The overall dispersal was prevalently upstream (Figure 9). On average, the dispersal direction was upstream in both generations and sexes (Figure 10); the distance travelled was dependent on generation (*p* < 10^−3^) but not dependent on sex (*p* = 0.22) and the sex*generation interaction (*p* = 0.85)—the summer generation individuals tended to disperse further upstream than the overwintered generation (Figure 10). Altogether, 58 females and 43 males, captured before overwintering, were recaptured upstream after the winter, whereas 26 females and 18 males either stayed or moved downstream during winter. The number of adults of both sexes that moved upstream during overwintering was significantly higher than those that stayed or moved downstream (χ^2^ = 10.25, *p* = 0.001 for males and χ^2^ = 12.19, *p* < 0.001 for females).

#### Remarkable Individual Records of *V. caprai*

Individual records revealed a long lifespan in *V. caprai*. Three individuals (two females, one male) marked in October 2008 were recaptured in April and May 2010, respectively, surviving at least two winters as adults. Moreover, thirteen other individuals (seven females and six males), marked in April 2009, were recaptured during spring and summer 2010, also surviving two subsequent winters as adults. Eight females, who moved upstream during overwintering, were found copulating after the winter.

## 4. Discussion

This long-term survey brought a unique view on dispersal patterns of semiaquatic bugs. Based on the results from solitary fishponds, it can be concluded that particular species differ in their dispersal abilities. The most convincing results are for *A. paludum*, *G. lacustris*, *G. argentatus*, and *G. odontogaster*. Whereas *G. odontogaster* and *G. argentatus* tend to stay at their sites and likely do not move much, *G. lacustris* is more mobile and moves along the shore, as a high proportion of *G. lacustris* movements were by one sector. Interestingly, *A. paludum* is also highly mobile, but moves across the open water rather than along the shore, as the highest proportion of the movements were diagonally across the fishpond (Figure 1). This is in accordance with habitat preferences found by [5], who stated that *G. argentatus* prefers littoral vegetation near the shore, *G. lacustris* prefers floating vegetation further from the shore, and *A. paludum* prefers open water furthest from the shore. Records for *G. thoracicus* and *L. rufoscutellatus* are scarcer and their moves are rather stochastic, thus their dispersal on the fishpond is quite unpredictable.

The comparison of dispersal among different wing morphs in males and females seems to be very important. The striking result is the significantly higher number of movements by flightless females compared with macropterous females and all males, probably general in Gerridae (Figure 2). It appears that flightless females compensate for their inability to fly (dispersal via air) by increasing their dispersal on the water surface. This brings a new dimension to the problem of the trade-off between dispersal capability and reproduction in wing dimorphic insects. Generally, macropterous females have lower reproductive output compared with flightless females, as compensation for their higher dispersal capabilities [13]. This also holds for Gerridae [14,15,16,17,18,19]. However, according to the data presented in this paper, the flightless females may have changed the dispersal behavior and disperse differently than via air. This would be especially advantageous in large or connected water bodies such as lakes, where flightless females may have higher reproductive output when demonstrating high dispersal capabilities. The result that reproductive flightless females disperse more than non-reproductive ones (Figure 4) supports this finding. This should hold especially for gerromorphan bugs, dispersing on the water surface, as the locomotion on the water surface is probably easier and faster than terrestrial insects. Similarly, water striders *A. najas* (a wingless species) from large and connected habitats move more than others from fragmented habitats [20]. A difference in water-surface movement between macropterous vs. flightless bugs could also be caused by a weight difference—the movement across the water surface could be more demanding for heavier individuals. Indeed, it seems that macropterous individuals tend to be heavier than brachypterous individuals [21], but the differences in particular species were not significant.

Interestingly, flightless males included in this research do not disperse more (on the water surface) compared with the macropterous ones. The reason for this may be that the trade-off between reproduction and flight is not well pronounced in males [13].

The change in the proportion of macropterous individuals, higher in marked vs. recaptured ones (Figure 5), confirms that macropterous individuals of all focal wing-dimorphic species migrate from the sampling site. The emigration from the fishponds is probably mostly during the flight to and from the overwintering site (flights of category 1 according to [7]), as the proportion of marked macropterous individuals significantly decreased during the winter in all wing-dimorphic species (Figure 5). The emigration of macropterous gerrids after winter is relatively common in both sexes of all species, except for *G. argentatus* males (Figure 5), but the sample size is relatively low in this case, and the philopatry of *G. argentatus* males cannot be confirmed.

The survey of interpond movements sorted out the species into three groups according to their dispersal pattern: (1) pond philopatric *A. paludum*, *G. lacustris* and *G. argentatus*; (2) sex-dependent philopatric *G. odontogaster* (females rather philopatric, males quite dispersive); (3) dispersal species (*G. thoracicus*, *G. gibbifer* and *L. rufoscutellatus*) (Figure 6 and Figure 7). Nevertheless, even the philopatric species can disperse from one fishpond to another, even repeatedly, but the proportion of dispersing individuals is lower. Sex-dependent dispersal of *G. odontogaster* can be explained by the sexual dimorphism in this species—males of this species force copulation using two abdominal processes [22]; such sexual dimorphism is lacking in other species in this survey. The aggressive males were shown to disperse more in another water strider, *A. remigis* [23], but the relationship between aggressiveness and dispersal can be present even in species comparison. The individual records also showed a high dispersal rate of *G. thoracicus*, *G. gibbifer* and *L. rufoscutellatus*. The recaptures of *G. lacustris* and *L. rufoscutellatus* (both males) after at least 6 km flight show the good dispersal abilities of these species, although the random transport by wind cannot be excluded.

The small pool systems are not suitable for any of the observed species—it appears that the pools attracted some individuals during migration from the overwintering site, but they recognized the site as not being appropriate for breeding and left for elsewhere. Interestingly, recaptures at the small pool systems showed the importance of vegetation for all species, as the individuals captured on the vegetated pools tended to stay (if recaptured), and those captured from bare pools tended to move. The vegetated pools might provide more prey, as some insects feeding on the plants can fall onto the water surface and be attacked by the water striders. Additionally, at least some preference of vegetated waters seems to be common for all species included in this study [24]. The short-term stay at these sites can be interpreted as (1) a possibly adaptive stay at the feeding site, before leaving to the breeding site. This could decrease the competition for food at the breeding sites. Potentially, the stay at these sites can also be (2) non-adaptive, a failure in finding a suitable site for reproduction during the post-overwintering flight.

The recaptures show a tendency to move upstream in *V. caprai*. This tendency was present in both males and females and both overwintered and summer generations. This supports the compensatory upstream dispersal, as predicted by [25]. However, the tendency to move upstream alone is not sufficient to conclude compensatory upstream dispersal [26,27]. Nevertheless, the upstream dispersal in *V. caprai* was present even during overwintering, and some of the overwintered females, which moved upstream during winter, were found copulating in the spring. It thus can be assumed that adults of *V. caprai* compensate for the downstream drift by active upstream dispersal. This upstream dispersal is probably terrestrial, as the sites on the forest streams were separated by riffles and weirs, making it difficult to travel on the water surface (Figure S13). The terrestrial movement of *V. caprai* was already described in [11]. This survey also confirms the unusual longevity of *V. caprai* and the ability to survive two winters as adults (and maybe one more as an overwintering egg, see [11,12,28]).

## 5. Conclusions

With regard to the dispersal pattern, the water strider species included in this survey differ from each other. After overwintering, all species migrate to a suitable breeding site (category 1 in [7] and the flights recorded in [8]), albeit with a facultative stop at the feeding site to avoid competition for food. After reaching the breeding site, *A. paludum*, *G. lacustris*, and *G. argentatus* tend to stay (with a small proportion of macropterous individuals dispersing elsewhere). Nevertheless, whereas *G. argentatus* is philopatric, *A. paludum* and *G. lacustris* disperse on the water surface all over the pond. Reproductive flightless females are predominant dispersers, *A. paludum* disperses over the open water, and *G. lacustris* disperses along the shore. A strong sex-dependent dispersal pattern is present in *G. odontogaster*—females are rather philopatric, with males often flying to nearby sites. The rest of the surveyed species, *G. gibbifer*, *G. thoracicus* and *L. rufoscutellatus*, are highly dispersing species, flying between ponds (category 2 in [7]). The only lotic species included in this survey, the water cricket *V. caprai*, tends to move upstream to compensate for the downstream drift.

## Figures and Tables

**Table 1:** Summary of marked individuals and catches at Kamenny fishpond (K) and Zavratsky fishpond (Z). Data pooled from all seasons.

Species	Fishpond	Marked Individuals	Population Proportion	Recapture Rate	Mean of Movements
*A. paludum*	K	791	0.23	0.25	1.65
	Z	1023	0.32	0.26	1.7
*G. lacustris*	K	1278	0.37	0.3	0.81
	Z	1215	0.38	0.31	0.76
*G. argentatus*	K	604	0.18	0.25	0.36
	Z	669	0.21	0.28	0.36
*G. odontogaster*	K	415	0.12	0.23	0.45
	Z	258	0.08	0.28	0.43
*G. thoracicus*	K	99	0.03	0.18	1.33
	Z	---	---	---	---
*L. rufoscutellatus*	K	234	0.07	0.16	1.82
	Z	20	0.01	0	---

**Table 2:** Statistical significance of the effect of sex, wing morph and their interaction on intra-pond movement score. Data pooled from all seasons and both fishponds. NS = non-significant; *** = *p* < 0.001.

Species	Sex	Wing Morph	Sex × Wing Morph
*A. paludum*	***	***	***
*G. lacustris*	***	***	***
*G. argentatus*	***	***	***
*G. odontogaster*	NS	***	NS
*G. thoracicus*	NS	------	------
*L. rufoscutellatus*	NS	------	------

**Table 3:** Statistical significance of effect of reproductive state, wing morph and their interaction on intra-pond movement score in females of *A. paludum* and *G. lacustris*. Data pooled from all seasons and both fishponds. * = *p* < 0.05; *** = *p* < 0.001.

Species	Reproductive State (RS)	Wing Morph	RS × Wing Morph
*A. paludum*	***	***	*
*G. lacustris*	***	***	***

**Table 4:** Summary of marked individuals and catches at Bily Kamen fishponds system (B) and Motovidlo fishponds system (M). Data pooled from all seasons.

Species	Fishpond System	Marked Individuals	Population Proportion	Recapture Rate	Mean of Movements
*A. paludum*	B	1896	0.32	0.25	0.25
	M	1962	0.33	0.26	0.19
*G. lacustris*	B	1987	0.33	0.24	0.29
	M	1920	0.32	0.26	0.31
*G. argentatus*	B	802	0.13	0.22	0.03
	M	924	0.15	0.32	0.04
*G. odontogaster*	B	672	0.11	0.35	0.9
	M	785	0.13	0.3	0.98
*G. thoracicus*	B	357	0.06	0.11	2.2
	M	190	0.03	0.18	2.46
*G. gibbifer*	B	62	0.01	0.13	1.5
	M	251	0.04	0.13	2.52
*G. lateralis*	B	0	0	------	-------
	M	17	<0.01	0.12	0.5
*L. rufoscutellatus*	B	231	0.04	0.12	1.96
	M	26	<0.01	0.12	1.67

**Table 5:** Summary of marked individuals and catches at Cep I and Cep II small-pools systems. Data pooled from all seasons.

Species	Site	Marked Individuals	Population Proportion	Recapture Rate
*A. paludum*	Cep I	31	0.13	0.16
	Cep II	58	0.19	0.09
*G. lacustris*	Cep I	124	0.54	0.15
	Cep II	156	0.5	0.13
*G. argentatus*	Cep I	31	0.13	0.16
	Cep II	41	0.13	0.2
*G. odontogaster*	Cep I	15	0.07	0.27
	Cep II	23	0.07	0.13
*G. thoracicus*	Cep I	0	0	-----
	Cep II	21	0.07	0.05
*G. gibbifer*	Cep I	29	0.13	0.07
	Cep II	5	0.02	0
*L. rufoscutellatus*	Cep I	0	0	-----
	Cep II	5	0.02	0

## Data Availability

The data presented in this study are available within the article.

## Supplementary Materials

The following are available online at https://www.mdpi.com/article/10.3390/insects12110976/s1. Figure S1: An example of the individually marked macropterous *Gerris lacustris*; Figure S2: An picture of unmarked brachypterous *Gerris* lacustris; Figure S3: A picture of unmarked water cricket *Velia caprai*; Figure S4: An example of the marked water cricket *Velia caprai*; Figure S5: A position of the Zavratsky fishpond in the landscape; Figure S6: Detailed aerial view of the Zavratsky fishpond; Figure S7: A position of the Kamenny fishpond in the landscape; Figure S8: Detailed aerial view of the Kamenny fishpond; Figure S9: A position of the fishponds of the Bily Kamen fishpond system; Figure S10: A position of the fishponds of the Motovidlo fishpond system; Figure S11: An aerial view of the Cep I small pool system; Figure S12: An aerial view of the Cep II small pool system; Figure S13: A picture of part of the stream where *Velia caprai* was sampled.
