# Supplementary material for: Dispersal and Migration Patterns of Freshwater Semiaquatic Bugs
Source: Insects. 2021 Oct 28;12(11):976. doi: 10.3390/insects12110976 (PMC8624209; doi:10.3390/insects12110976)
Supplement: Supplementary file 1 [file insects-12-00976-s001.zip › insects-1379265-supplementary.pdf]

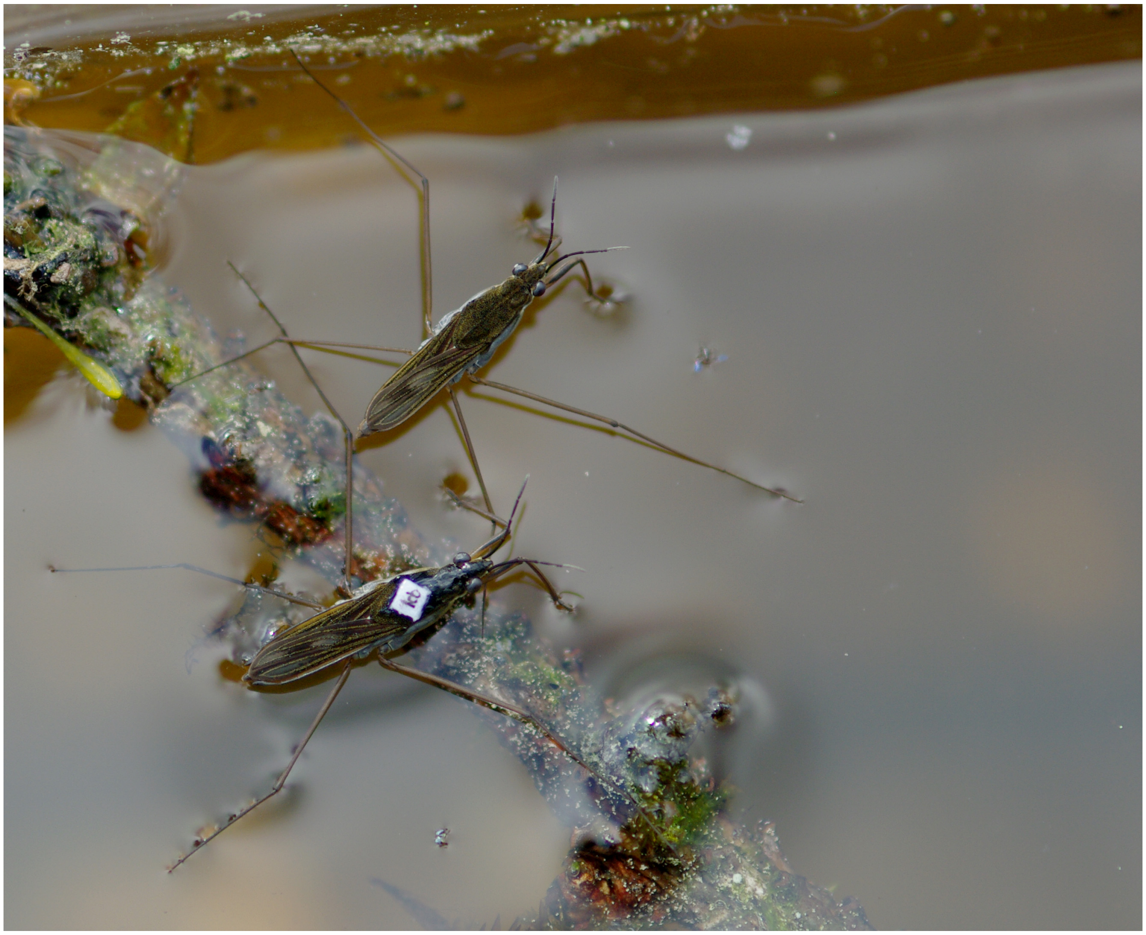

Figure S1. A marked (code *kb*) and unmarked macropterous adult of the water strider *Gerris lacustris* (Gerridae). Size of the *G. lacustris* adults is 8 – 10 mm.

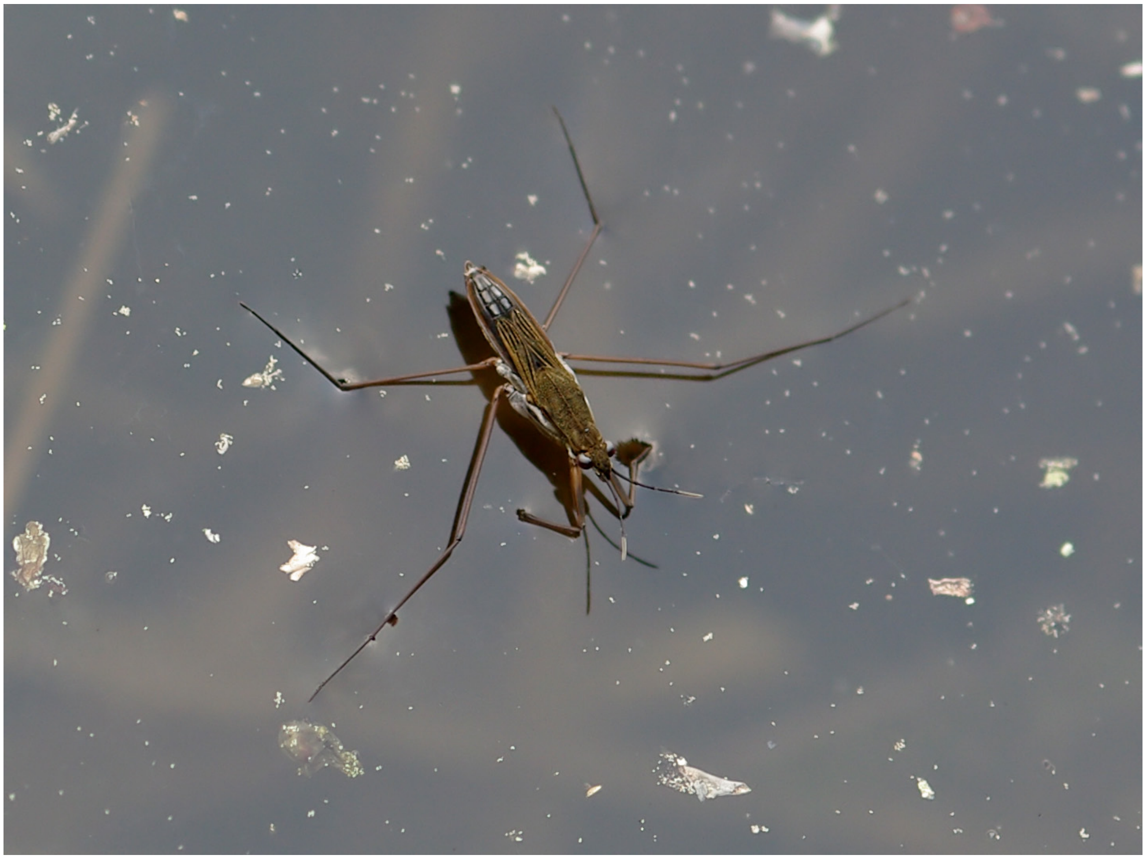

Figure S2. An unmarked, brachypterous adult of the water strider *Gerris lacustris* (Gerridae).

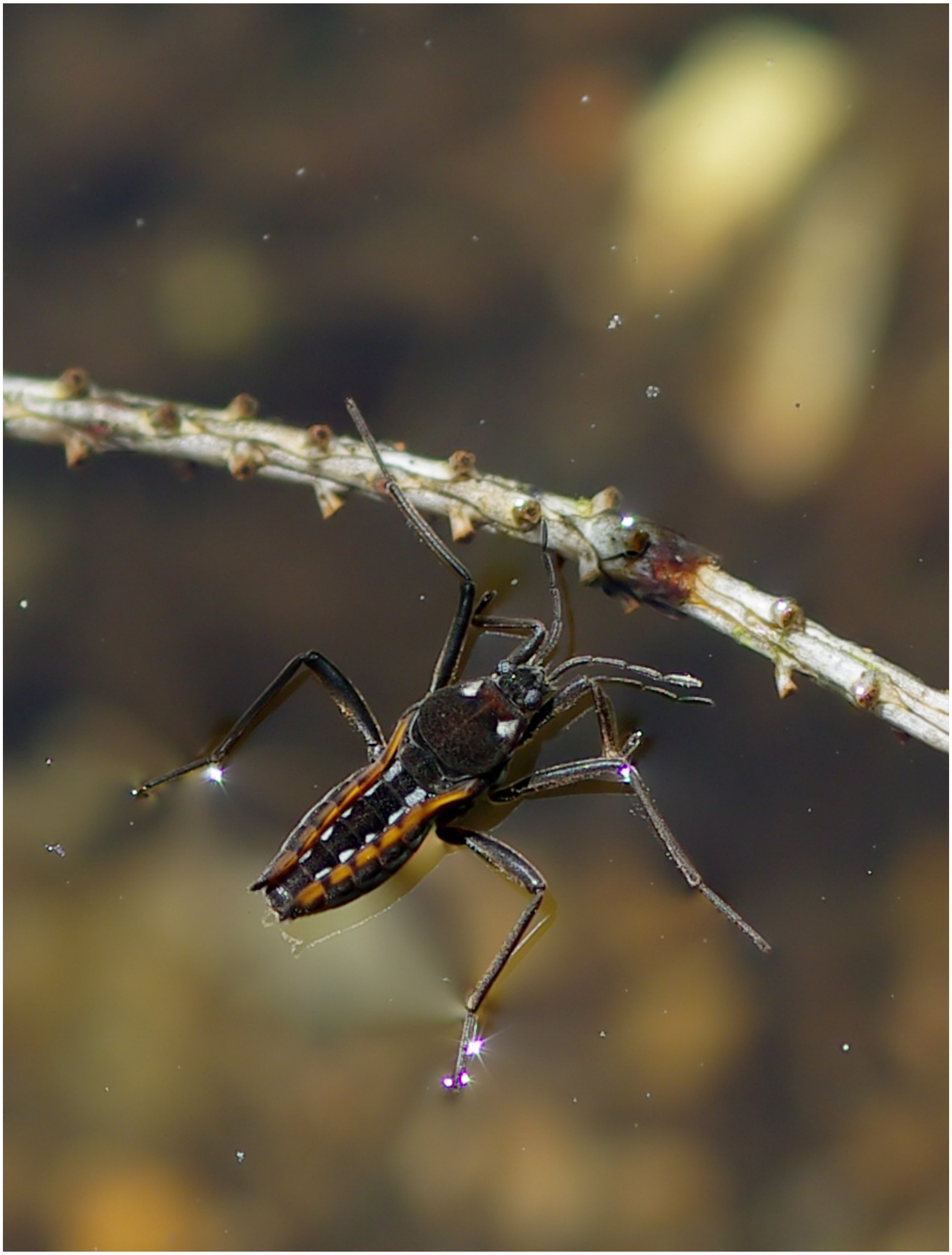

Figure S3. An unmarked adult of the water cricket *Velia caprai* (Veliidae). Size of the adults is 6 – 8 mm.

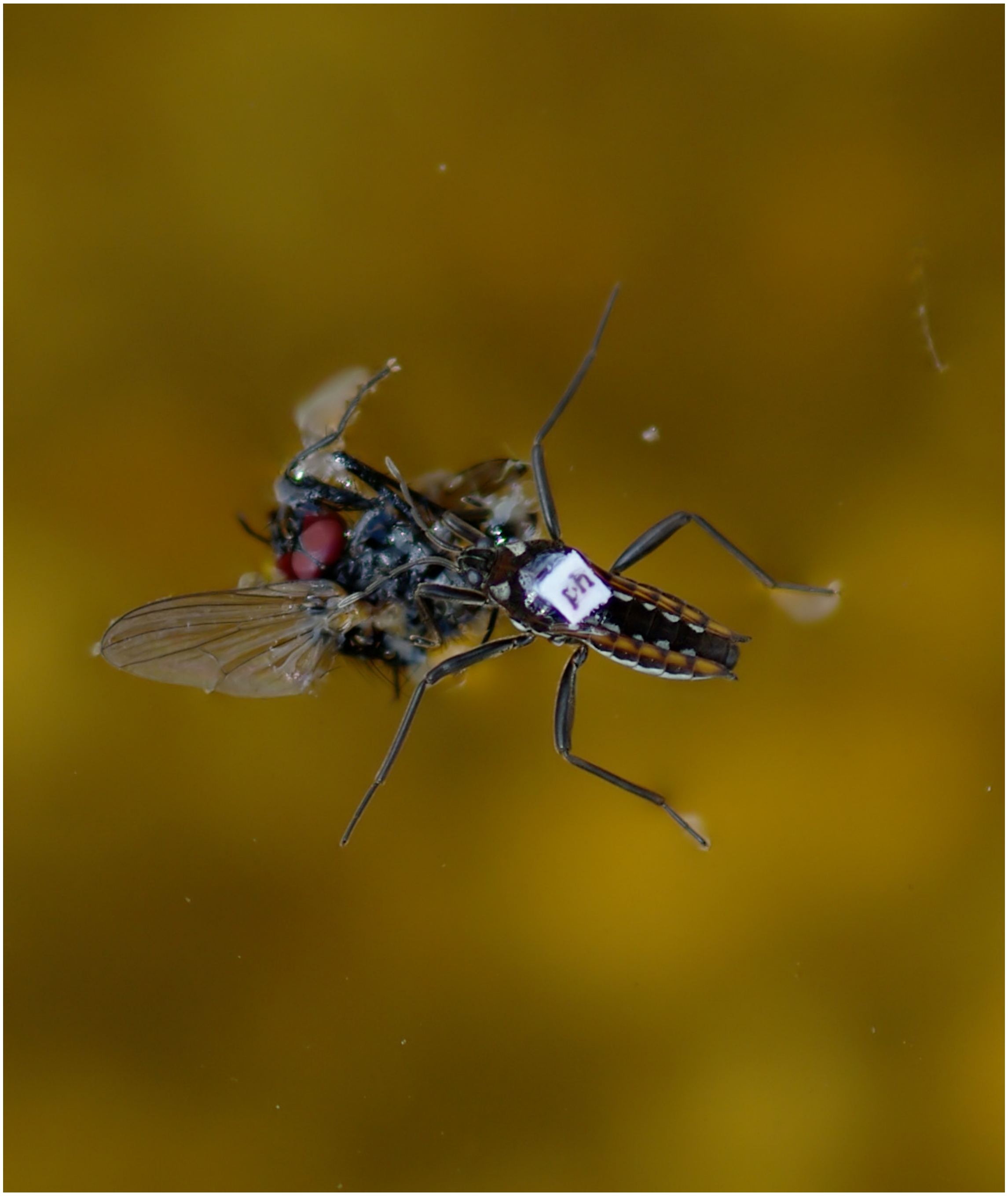

Figure S4. A marked adult of *Velia caprai* (Veliidae), feeding on a housefly (*Musca domestica*).

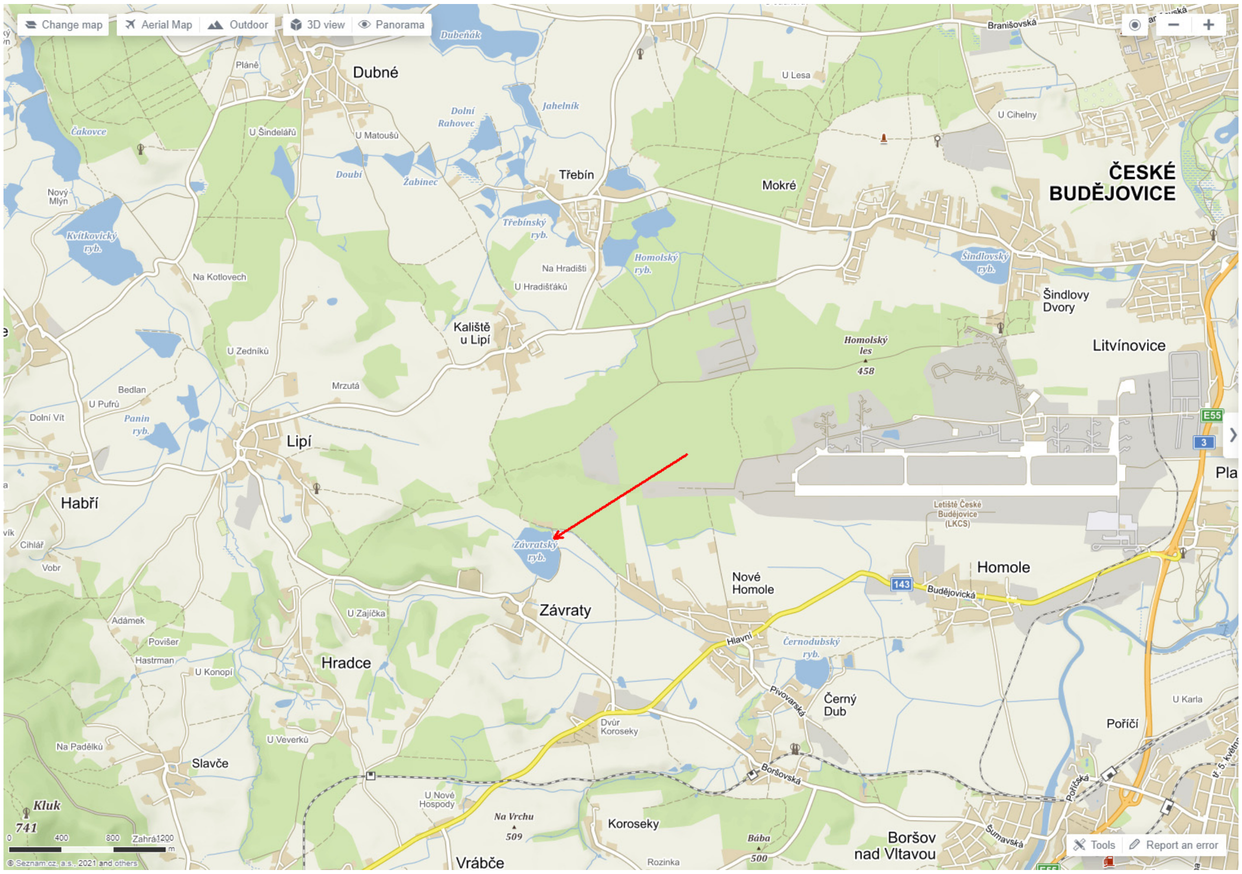

Figure S5. A position of the Zavratsky fishpond in the landscape (red arrow). It is located approx. 2.2 km far from the closest fishpond. Source of the map: "Mapy.cz, use permitted under the license CC-BY-SA 4.0. Online map can be accessed at https://en.mapy.cz/s/nodezolabe.

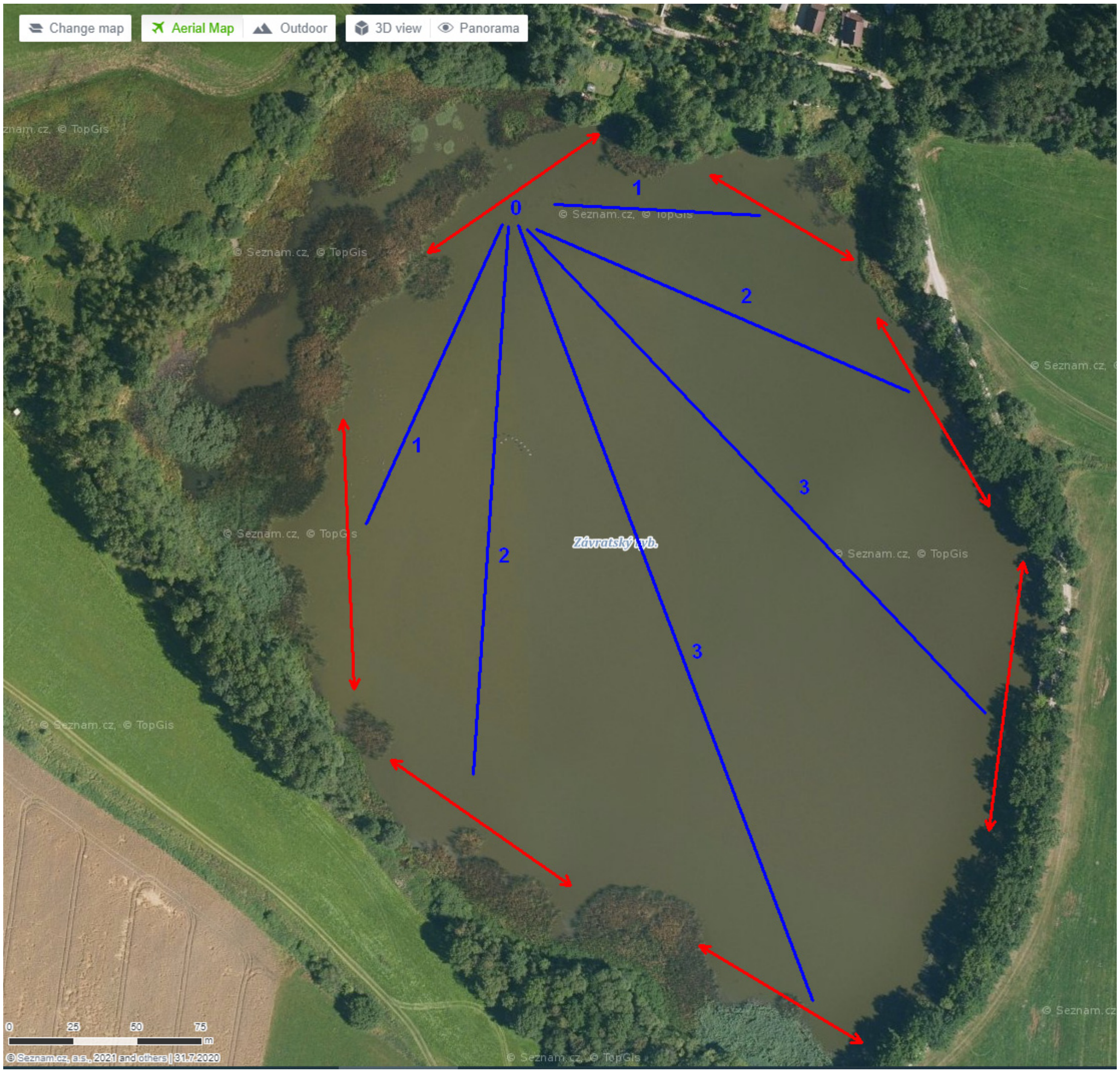

Figure S6. Detailed aerial view of the Zavratsky fishpond. The approximate sampling sites (sectors) are indicated by red arrows. Blue lines indicate the scoring of the movement of an individual, originally captured at the northernmost sector. Source of the map: "Mapy.cz, use permitted under the license CC-BY-SA 4.0.

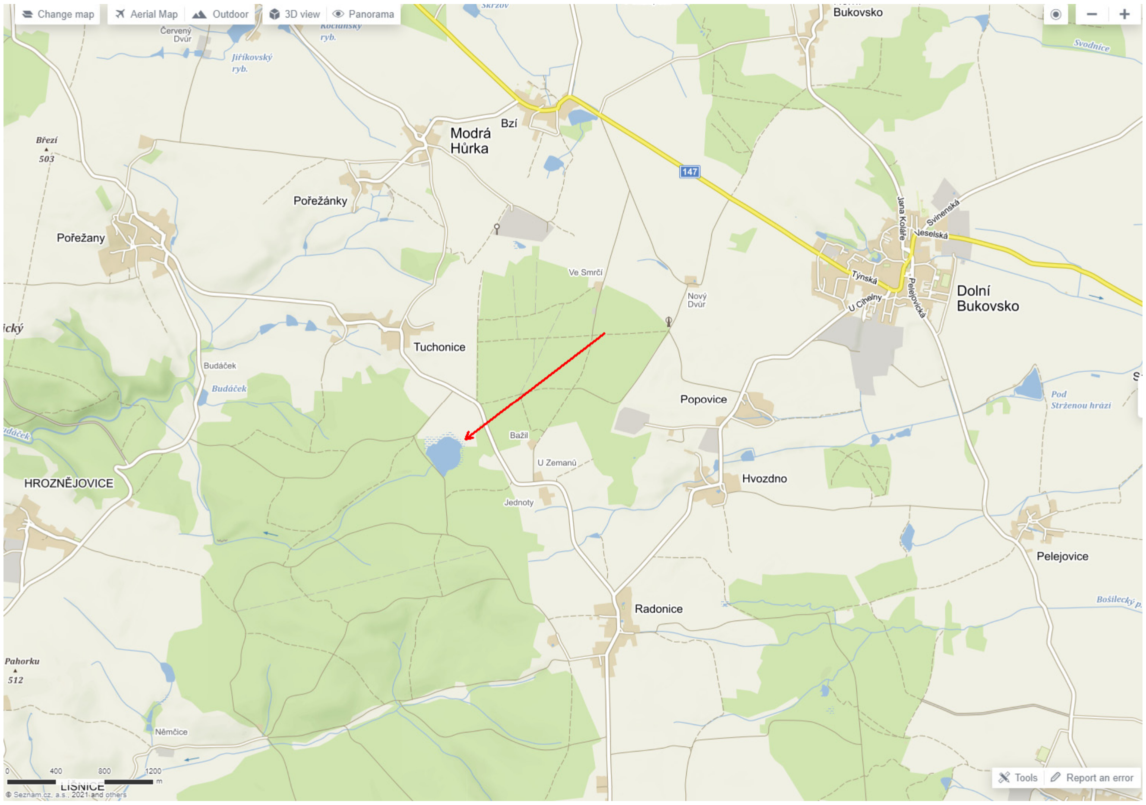

Figure S7. A position of the Kamenny fishpond in the landscape (red arrow). It is located approx. 1.6 km far from the closest fishpond. Source of the map: "Mapy.cz, use permitted under the license CC-BY-SA 4.0. Online map can be accessed at https://en.mapy.cz/s/bonujavase.

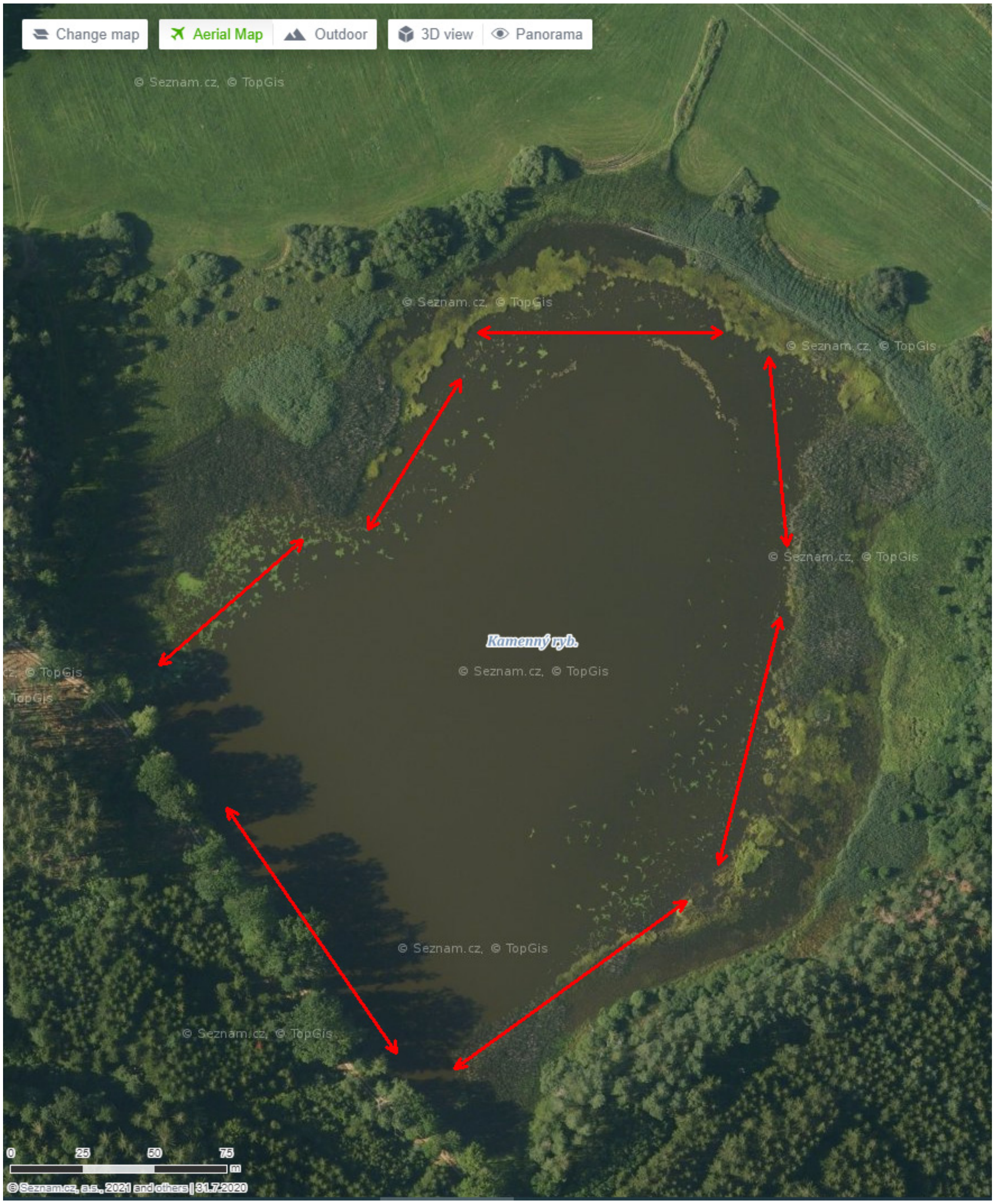

Figure S8. Detailed aerial view of the Kamenny fishpond. The approximate sampling sites (sectors) are indicated by red arrows. The scoring of the movement was alike as in the Zavratsky fishpond. Source of the map: "Mapy.cz, use permitted under the license CC-BY-SA 4.0.

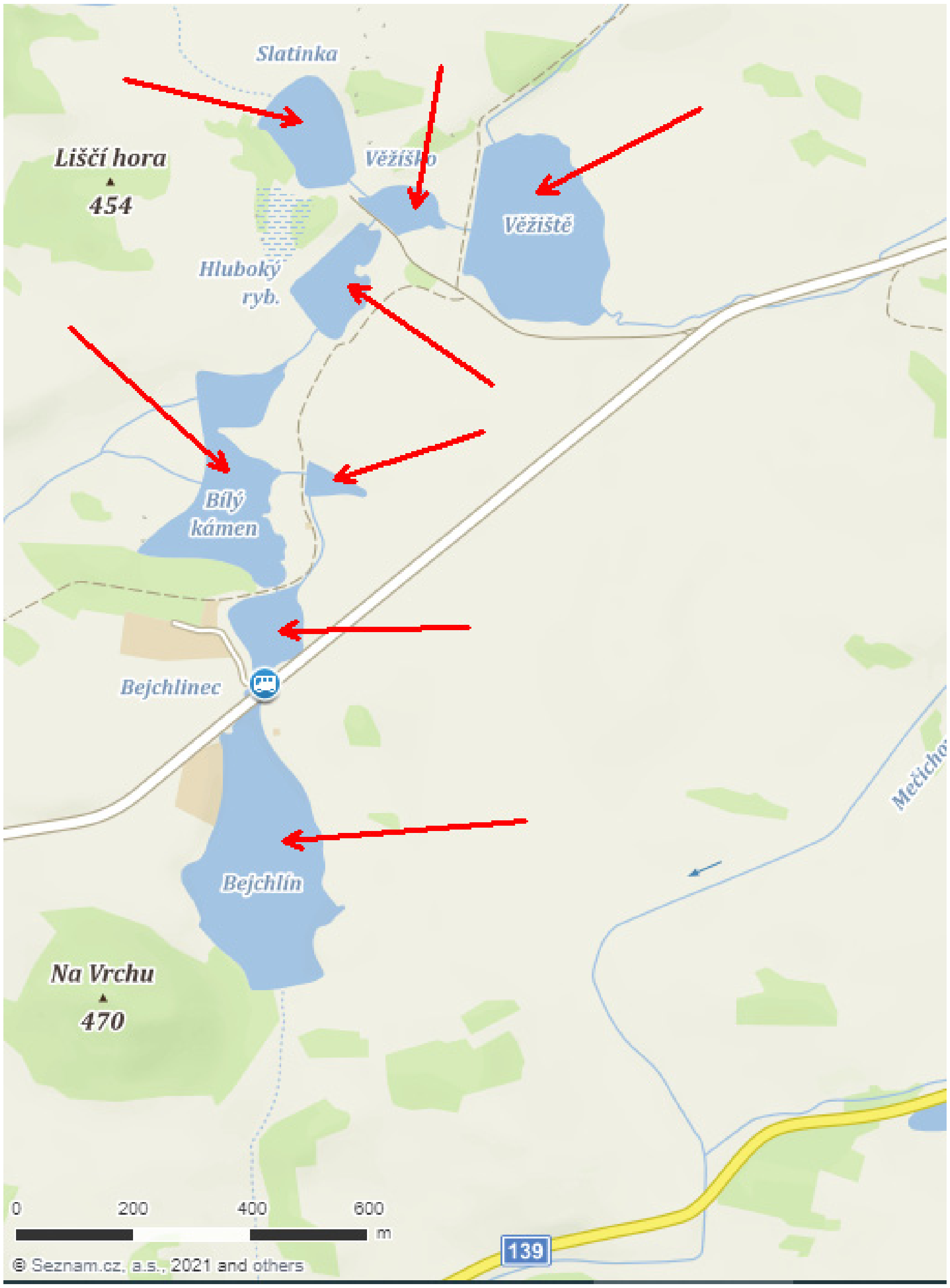

Figure S9. A position of the fishponds (red arrows) of the Bily Kamen fishpond system. Source of the map: "Mapy.cz, use permitted under the license CC-BY-SA 4.0. Online map can be accessed at https://en.mapy.cz/s/gebujujumu.

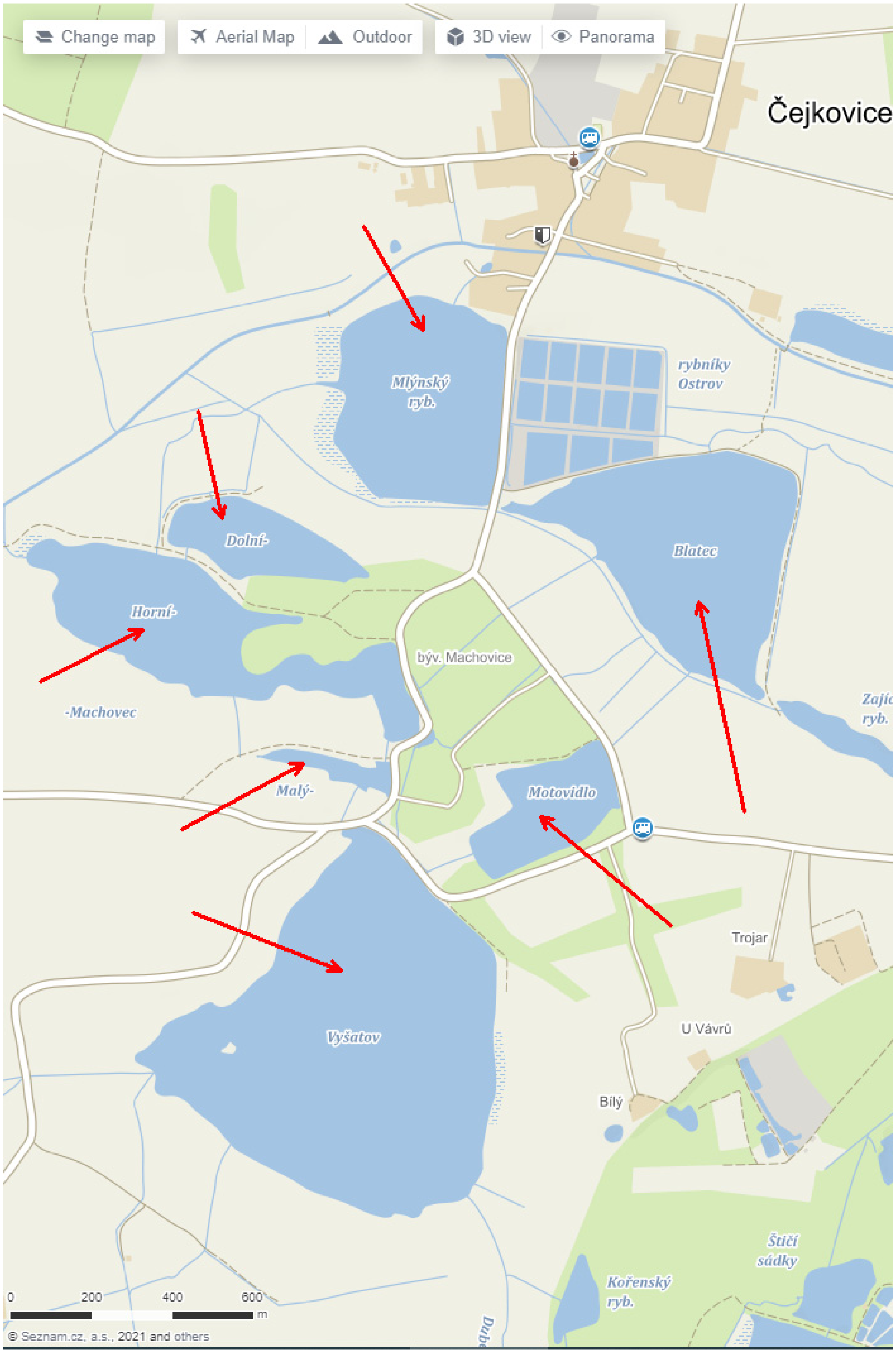

Figure S10. A position of the fishponds (red arrows) of the Motovidlo fishpond system. Source of the map: "Mapy.cz, use permitted under the license CC-BY-SA 4.0. Online map can be accessed at https://en.mapy.cz/s/pofurefoja.

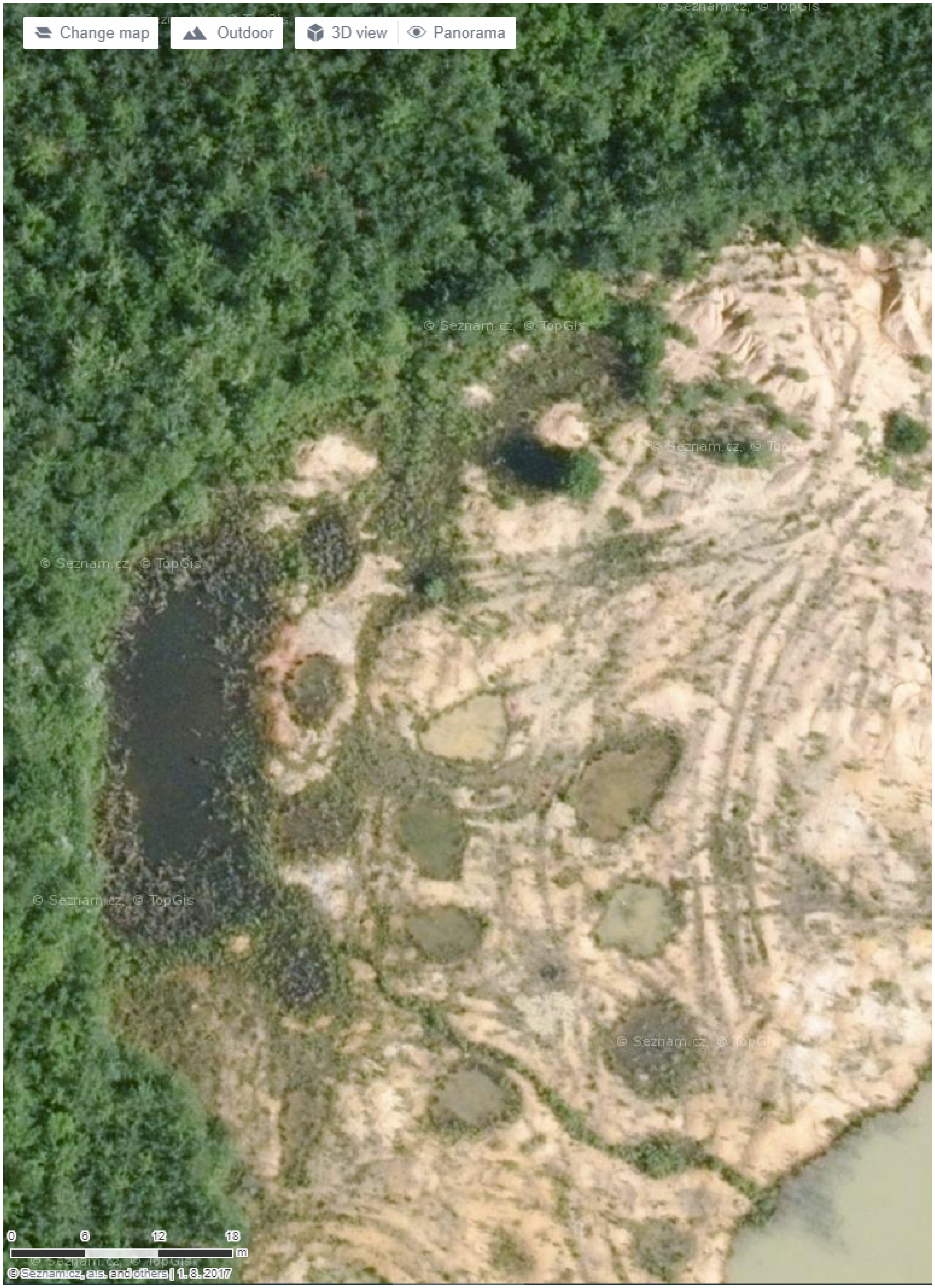

Figure S11. An aerial view of the Cep I small pools system. Source of the map: "Mapy.cz, use permitted under the license CC-BY-SA 4.0. Online map can be accessed at https://en.mapy.cz/s/cozozuhoju.

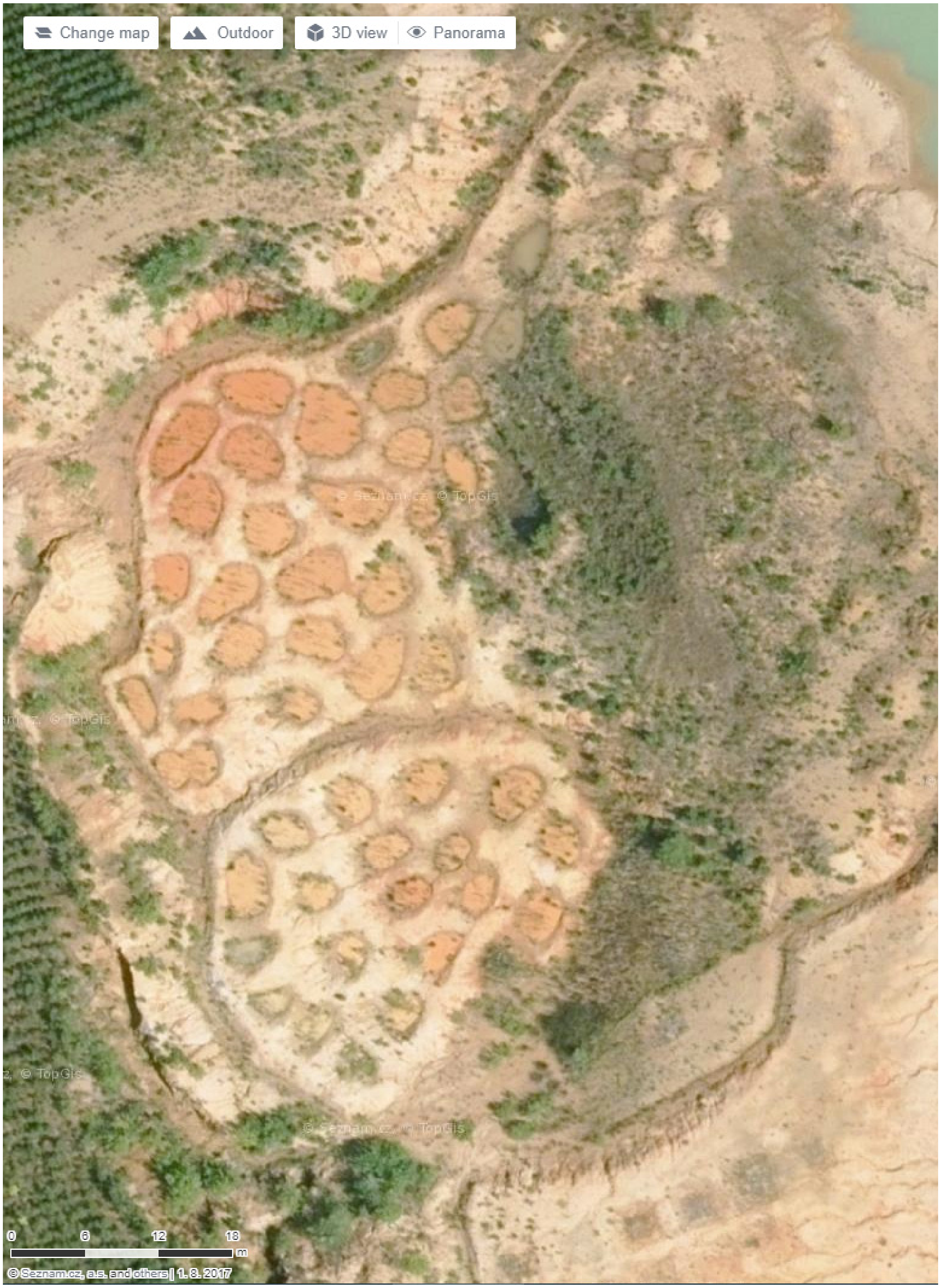

Figure S12. An aerial view of the Cep II small pools system. Source of the map: "Mapy.cz, use permitted under the license CC-BY-SA 4.0. Online map can be accessed at https://en.mapy.cz/s/dagedukace.

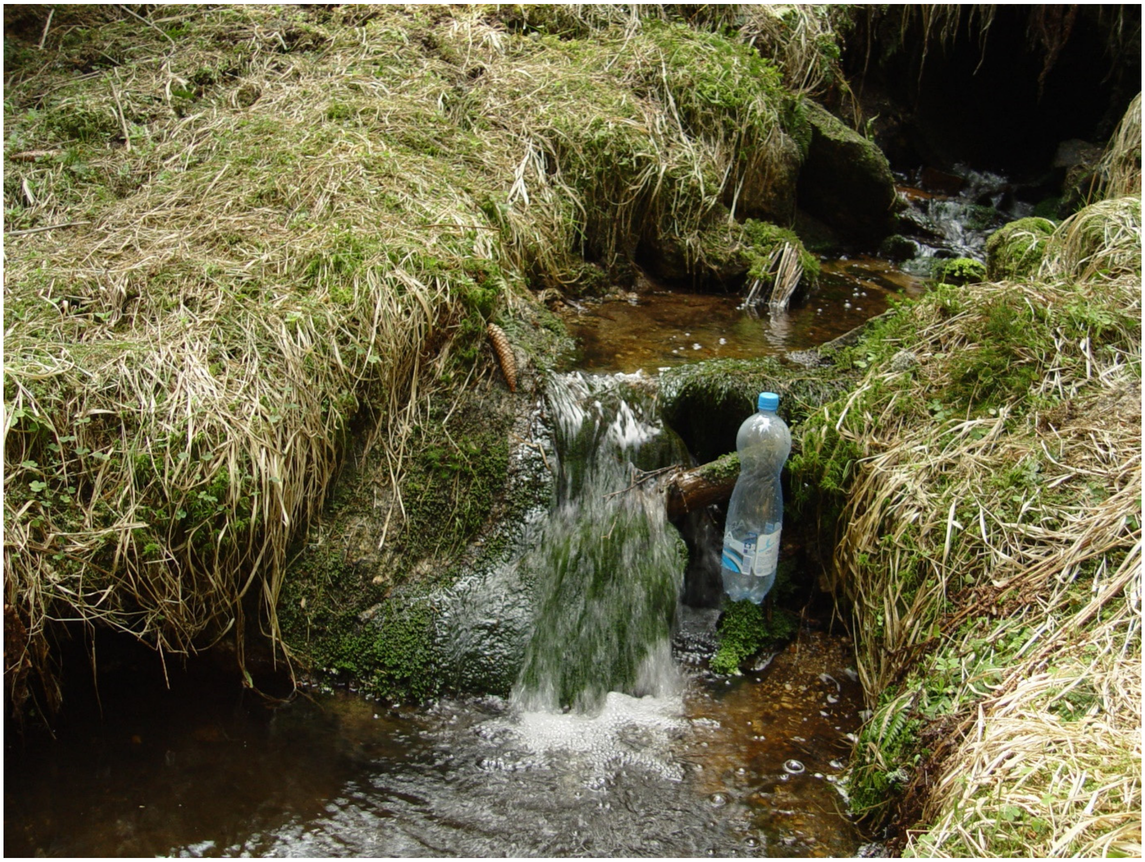

Figure S13. Part of the stream, where the water cricket *Velia caprai* (Veliidae) was sampled. The stream consists of the places with variable current velocity. Some weirs could not be passed via the water surface.
